# Role reconstruction and competency requirements of emergency nurses in the human-machine collaborative mode: a qualitative study

**DOI:** 10.3389/fpubh.2026.1831771

**Published:** 2026-04-23

**Authors:** Lina Chen, Qi Wang, Xinrui Mao, Shuqin Xia, Ao Ren, Lu Yin, Hang Wang, Na Liu

**Affiliations:** 1 Department of Emergency, Shanghai East Hospital, Shanghai, China; 2 School of Nursing, Hubei University of Medicine, Shiyan, China; 3 Shanghai East Hospital, School of Medicine, Tongji University, Shanghai, China; 4 School of Medicine, Tongji University, Shanghai, China

**Keywords:** competency requirements, emergency nurses, human-machine collaboration, qualitative study, role reconfiguration

## Abstract

**Objective:**

This study aims to explore the role reconstruction characteristics and capability requirements of emergency nurses in the human-computer collaboration mode, providing experience and reference for improving the quality of nursing.

**Methods:**

A phenomenological qualitative research method was adopted. Nurses from the emergency departments of two tertiary hospitals in Shanghai from October 2025 to January 2026 were selected as the research subjects for semi-structured interviews, and the Colaizzi seven-step analysis method was used for data analysis.

**Results:**

A total of 22 emergency department nurses participated in this study, and 3 major themes and 8 sub-themes were extracted. (1) Nurses’ cognition and attitude towards the human-machine collaboration model; (2) The role transformation of emergency nurses; (3) Nurse competency requirements.

**Conclusion:**

The results of this study clarify the role transformation and capability requirements of emergency nurses in human-machine collaboration, providing practical significance for clinical training, role boundary definition, workflow optimization and policy formulation, thereby promoting the standardized, safe and sustainable application of human-machine collaboration in emergency care.

## 1 Introduction

The emergency department is the frontline for treating critical and severe cases in hospitals, undertaking core functions such as rapid triage, emergency rescue, and multi-disciplinary collaboration. Its work is characterized by high pressure, high risk, high pace, and strong uncertainty (1, 2). As key practitioners in the emergency care process, nurses are directly involved in the entire continuum of care, from triage to discharge. Their professional competence and work efficiency directly influence the quality of emergency care and patient outcomes (3). In recent years, global demand for emergency medical care has continued to rise. Relevant studies indicate (4), that emergency departments are universally facing an increase in patient numbers, with an annual growth rate of 10%, leading to a widespread overload of work for emergency nurses globally. Traditional nursing models struggle to meet the demands of the evolving emergency care landscape (5). In China, with the rapid development of digital technologies such as artificial intelligence (AI), big data and the Internet of Things, human-machine collaboration models have gradually entered the healthcare sector. Through cognitive consensus and dynamic interaction, humans and intelligent systems form deep collaboration, effectively improving work quality and efficiency (6). Within the field of emergency nursing, human-machine collaboration has permeated every aspect of practice. From AI-powered emergency triage systems (7) and intelligent monitoring and alert devices (8) to intelligent decision-support systems for emergency care (9), a diverse range of applications has emerged. These innovations free nurses from tedious routine tasks and data recording, driving a shift in emergency nursing from an experience-driven model towards one of human-machine collaboration. Relevant studies have shown that (10) the human-machine collaborative mode can effectively enhance nursing efficiency and optimize work processes. During this transformation process, emergency nurses need to redefine their roles. Traditional emergency care mainly focuses on executive work. However, under the human-machine collaborative model, nurses have transformed from simple operators to multiple roles such as coordinating decision-makers, humanistic caregivers, and intelligent system detectors. Their daily work is shifting from manual data collection and experience-based judgment to supervising and validating AI recommendations, and making higher-level clinical decisions based on this. In the human-machine collaboration mode, machines have taken over some information processing and analysis tasks, but this also poses new challenges for emergency nurses. Although AI systems can enhance efficiency, they lack training in complex and atypical clinical scenarios, which may result in misleading outputs (11), requiring nurses to possess the ability to identify and evaluate AI-generated information. Furthermore, emergency nurses must balance humanistic care with ethical judgement to resolve potential doctor-patient conflicts that may arise within the human-machine collaboration model. In recent years, AI technology has developed rapidly, and scholars both domestically and internationally have conducted extensive research into human-machine collaboration in the emergency department. However, most of the existing research focuses on the application of tools such as artificial intelligence triage and intelligent detection, and there are few qualitative studies exploring nurses’ views and requirements regarding the role reconstruction process. As key practitioners in the clinical care process, the perspectives and needs of emergency nurses regarding this process of role reconfiguration will help to identify the facilitating and hindering factors in the transformation of their roles under human-machine collaboration models. Therefore, this study aims to use qualitative research methods to explore the views and needs of emergency nurses regarding role reconfiguration within human-machine collaboration models in the Chinese cultural context, thereby providing guidance for alleviating the pressure of role adaptation among emergency nurses and enhancing the quality and safety of emergency nursing care.

## 2 Methods

### 2.1 Study design and participants

This study employed a phenomenological qualitative research design, utilizing semi-structured in-depth interviews to explore the subjective experiences, role changes and competency requirements of emergency nurses working within a human-machine collaboration model. To ensure the accuracy of the research findings, purposeful sampling was used to select nurses from the emergency departments of two tertiary hospitals in Shanghai between October 2025 and January 2026 as the study participants. The research process strictly adhered to the COREQ guidelines for qualitative research reporting (12), ensuring the rigor, transparency and reproducibility of the study.

#### 2.1.1 Inclusion and exclusion criteria

Inclusion criteria: (1) Hold a valid nurse practice certificate and be engaged in front-line clinical nursing work in the emergency department; (2) Having been engaged in front-line clinical nursing work such as emergency pre-examination and triage, rescue, and emergency monitoring for more than 1 year, and confirmed by the head nurse to be capable of independently completing daily emergency nursing tasks; (3) Within the past 6 months, there has been practical experience in using human-machine collaboration systems or devices such as AI pre-examination and triage systems, intelligent monitoring, AI early warning systems, and intelligent decision-making assistance tools, and one can clearly describe their own experience, role changes, and ability difficulties in the human-machine collaboration process. (4) Voluntarily participate in this study, sign the informed consent form, and express oneself clearly in language, capable of completing in-depth interviews.

Exclusion criteria: (1) Non-emergency department nurses (such as ward nurses, outpatient nurses, operating room nurses); (2) Has not used the human-machine collaboration system/equipment in the emergency department; (3) Trainee nurses and standardized training nurses who have not yet started working independently; (4) Not having worked on the front line of emergency departments in the past 3 months (such as long-term leave, secondment); (5) Those who have communication barriers or are unable to cooperate with the interview.

#### 2.1.2 Determination of sample size

The sample size was determined based on the principle of data saturation, which is reached when no new themes, perspectives or experiences emerge during three consecutive interviews (13). A total of 28 emergency nurses who met the inclusion criteria were recruited in this study. Among them, 3 nurses were unable to cooperate to complete the interviews due to work schedule conflicts. Eventually, 25 nurses participated in the qualitative interviews. Prior to the formal interviews, pilot interviews were conducted with three of the emergency nurses. As the primary purpose of the pilot interviews was to test the scientific validity, rationality and feasibility of the interview guide, rather than to formally collect data (14), the interview content from these three participants was not included in the study results. From the 20th participant onwards, no new themes emerged during the interviews with the subsequent three participants; consequently, 22 emergency nurses were ultimately included in the study.

### 2.2 Data collection method

The research team established cooperation with the head nurses of the emergency departments of two tertiary hospitals in Shanghai, elaborating on the purpose, significance and process of this study. It was clearly stipulated that the head nurses would serve as recruitment assistants, responsible for the initial screening and communication of nurses within the hospital who met the inclusion criteria. A preliminary list of potential participants who meet the requirements will be provided to the research team. Researchers who have received qualitative research training formulate standardized recruitment notices, and the head nurse issues recruitment announcements through departmental meetings. If potential participants are willing to take part, they can contact the researchers proactively. The researchers will conduct a second qualification check on them one by one to confirm whether they fully meet the inclusion and exclusion criteria. Participants who pass the inspection will be listed as candidate interviewees. Before the formal interview, the purpose and significance of this study were introduced to the participants, and their willingness was sought to sign the informed consent form. Subsequently, we collected general sociodemographic information, including age, gender, educational background, years of emergency work experience, professional title, specific job position, duration of use of human-machine collaborative systems, frequency of use of human-machine collaborative systems, and commonly used equipment/systems. These data are crucial for analyzing the role reconfiguration and capability requirements of emergency nurses in the human-machine collaboration model. Finally, participants were interviewed in a one-on-one structured format in a quiet meeting room. The interview process was conducted by two researchers who had received qualitative research training. With the consent of the respondents, the interview process was recorded. In addition, the researchers observed and recorded the language and expressions of the respondents. In view of the respondents’ vague expressions and implicit emotional expressions, the researchers used open-ended and exploratory questions to explore the respondents’ deep emotional experiences. The entire interview process lasted from 26 to 49 min. The content of the interview will be transcribed within 24 h after its conclusion. After the transcription is completed, the corresponding transcribed text content is returned by the researcher to the corresponding interviewee for inspection and verification, with a focus on confirming whether the interview content is complete, whether the expression is accurate, and whether there is any transcription bias. This process allows respondents to make corrections, additions or deletions. After receiving the verification feedback from the respondents, the research team conducted the final calibration of the transcribed text.

### 2.3 Interview guide

According to the research purpose, a semi-structured interview guideline was compiled. To further enhance the scientific, pertinence and feasibility of the interview guidelines, during the compilation process, we invited five experts to participate in the consultation. Among them, three are head nurses of the emergency department with over 10 years of experience in emergency nursing, one is a qualitative research expert who has long been engaged in qualitative research, and one is an expert in the field of AI who has long been involved in research in the intersection of medicine and engineering. The research team sent the initial interview guidelines to five experts online, asking them whether the content of the interview Outlines was comprehensive. Is there any information that deviates from the topic? Does the expression of the interview outline accurate? Subsequently, five experts independently put forward revision suggestions. After summarizing the experts’ opinions, the research team held an online group meeting to discuss and revise the existing problems (such as some problems being misleading and some problems not closely related to the theme). The revised interview guidelines were sent again to five experts for verification, and none of them put forward any new opinions. Then, pre-interviews were conducted with the three recruited participants, and the logical sequence of the interviews was adjusted, and the clinical scenarios were supplemented based on their feedback. The core issues of the final interview guideline are shown in Table 1.

**Table 1:** Interview guide.

Theme	Question
Basic experience	1. Which AI devices have you used in your emergency work? What is the duration and frequency of use? How stable is it?
	2. How was your experience during the usage process? What are the advantages or what difficulties have been encountered?
Role change	3. What changes have occurred in your daily work content after using human-machine collaborative devices? How have these changes affected your perception of your role?
	4. In your opinion, what changes have occurred in the role of emergency nurses under the human-machine collaboration mode compared with the traditional mode?
	5. Have you ever encountered difficulties in adapting to your role during the process of character change? If so, what are the specific manifestations?
	6. What do you think are the main factors influencing the role changes of emergency nurses?
Competency requirements	7. What core capabilities do you think emergency nurses need to possess to efficiently adapt to the human-machine collaboration mode?
	8. Compared with traditional emergency nursing work, in the human-machine collaborative mode, in which aspects do you think you are lacking? How have these deficiencies affected your work?
	9. What kind of support do you hope the hospital or relevant departments will provide to help emergency nurses better complete their role changes and enhance their ability to adapt to the human-machine collaboration mode?
Others	10. Do you have any other information that needs to be supplemented?

### 2.4 Research team

The research team consists of 6 experienced nursing experts and 2 postgraduate nursing students. All team members have received methodological training in qualitative research and are familiar with the COREQ guidelines and research procedures. The research team has a clear division of labor. The first author and the second author are, respectively, a graduate student in nursing and a clinical expert, who were responsible for the research design, interviews and data analysis. During the interview, the two researchers asked questions and made observation records, respectively. During the research process, the research team held group discussions every week to synchronize the research progress and jointly discuss the problems that emerged during the research process. If no consensus was reached, the decision was made by the corresponding author, Na Liu, who is an expert in the field of emergency medicine and has rich professional knowledge and clinical experience in this field to ensure the scientific and accuracy of the research process.

### 2.5 Data analysis method

Import the transcribed text into the NVivo 15.0 software for analysis. Data analysis was conducted using the Colaizzi seven-step analysis method. The specific analysis steps include familiarizing with the data, extracting meaningful statements, encoding the meaningful statements, categorizing and organizing the encoded statements to extract the theme, providing a detailed description of the theme, returning to the research object for verification, and forming the results (15). To enhance the rigor of the research process, the analysis and coding process is independently completed by two researchers. If there are any differences between the two researchers, the first researcher will repeatedly discuss and resolve them until a consensus is finally reached. Subsequently, the former themes and detailed descriptions are fed back to the original participants, asking them whether the content of these themes is in line with their real experiences and cognition, judging whether the themes are accurate and comprehensive, and offering suggestions for supplementation or modification. This process is completed within 10 days after the entire data analysis is finished to ensure that the research subjects have a clear memory of the interview content. All the participants believed that these topics were in line with their viewpoints.

### 2.6 Rigor

This study strictly adhered to the COREQ guidelines for qualitative research (12). To ensure the scientific and reliable nature of the entire research process, the research team made a reasonable division of labor and carried out specialized training before recruiting participants. Before the interview, the time and place of the interview were reasonably arranged, and the trust of the interviewees was gained. During the interview, the researcher maintained a neutral stance throughout and made no suggestive or misleading remarks. Throughout the entire research process, reflective practice was relied upon to enhance the credibility of the research. After each interview, two researchers wrote interview notes, focusing on recording potential biases that might exist during the implementation of the interviews. They proactively disclosed and jointly analyzed these biases in the regular discussions of the research group, and promptly formulated improvement measures to avoid the impact of biases. Meanwhile, to further enhance the reliability of the research, this study adopted a tripartite adjustment approach involving researcher analysis, verification of research subjects, and expert evaluation, minimizing the interference of various biases on the research results to the greatest extent.

### 2.7 Ethical considerations

This study has been approved by the Medical Ethics Committee of Shanghai East Hospital Affiliated to Tongji University (2025–095). Before the formal interview, the researcher fully informed the participants of the purpose, significance, process, potential risks of this study and the rights of the research subjects, including the right to voluntarily participate, the right to withdraw at any time without any adverse effects, and the right to personal privacy protection. All the research subjects signed a written informed consent form before participating in the interview. All audio recordings and written records are for research and analysis purposes only and may not be used for any other purposes without the permission of the participants.

## 3 Result

A total of 22 eligible emergency department nurses participated in this study (Table 2). Among them, there are 4 males and 18 females. The participants’ ages ranged from 23 to 45 years old, with an average age of 31.59 years old. The working years in the emergency department range from 1 to 21 years, with an average of 8.00 years. In terms of educational attainment, 16 people have a bachelor’s degree or above, accounting for 72.73%. In addition, the frequency of using human-machine collaborative devices is daily, and the usage period is 0.5 to 3 years. Commonly used devices include AI triage systems, central monitoring systems, and mobile nursing PDAs. Ultimately, three themes and eight sub-themes were extracted through interviews, as shown in Table 3.

**Table 2:** General information of respondents.

Variable	Type	Frequency (n)	Percentage (%)
Age (year)	< 30	9	40.91
	30 ~ 40	10	45.45
	> 40	3	13.64
Gender	Man	4	18.18
	Woman	18	81.82
Educational level	Junior college	6	27.27
	Undergraduate	13	59.09
	Master’s degree	3	13.64
Professional title	Junior	15	68.18
	Intermediate	7	31.82
Emergency working years	< 3 years	3	13.64
	3 ~ 5 years	7	31.82
	6 ~ 10 years	5	22.73
	> 10 years	7	31.82
Job position	Triage	5	22.73
	Resuscitation room	6	27.27
	Infusion room	4	18.18
	Intensive care unit	4	18.18
	Emergency ward	3	13.64
Usage time of AI devices	< 1 years	2	9.09
	1 ~ 2 years	13	59.09
	> 2 years	7	31.82

**Table 3:** Summary of main themes and subthemes.

Main themes	Subthemes
Emergency nurses’ cognition and attitude towards human-machine collaboration.	1. The HMC Mode can alleviate the work pressure of nurses and improve their work efficiency.
	2. Nurses hold a cautious attitude towards the human-machine collaboration model.
Role transformation of emergency nurses in HMC mode.	1. Transform from an experience-based executor to an AI collaborative decision-maker.
	2. The humanistic care role of emergency nurses has been enhanced.
	3. The boundaries of the nurse’s role are blurred.
Competency requirements of emergency nurses in human-machine collaboration mode.	1. The demand for critical thinking ability in recognizing information output by AI.
	2. The demand for intelligent device operation and emergency handling capabilities.
	3. The need for awareness of data security and ethical risks.

### 3.1 Theme 1: emergency nurses’ cognition and attitude towards human-machine collaboration

Emergency nurses generally recognize that human-machine collaboration can reduce workload and improve diagnosis and treatment efficiency, but they still hold a cautious attitude in clinical application, paying attention to the reliability and safety of the technology.

#### 3.1.1 The HMC mode can alleviate the work pressure of nurses and improve their work efficiency

The application of the HMC Mode in emergency nursing practice has effectively reduced the work pressure of nurses and significantly improved the clinical work efficiency. Most nurses indicated that the HMC model’s intervention in pre-examination and triage, information verification, risk warning and other links has reduced repetitive and cumbersome basic operations, enabling nurses to have more time to focus on the disease changes of patients and improve medical quality. In the high-intensity and fast-paced working environment of the emergency department, the auxiliary role of AI tools has effectively alleviated the time pressure and workload of nurses, and reduced the anxiety caused by information omissions and cumbersome procedures. Meanwhile, the rapid identification and warning of key risks by AI help nurses identify potential critically ill patients earlier, improve the response speed of treatment, and further enhance their sense of control over work and professional efficacy.

*N6: In the past, we had to ask questions ourselves for triage. During peak hours, the queue was long and the more anxious we were, the more likely we were to make mistakes. Nowadays, the AI-assisted triage system can help us with triage based on the chief complaint and vital signs, saving the time for triage.*

*N12: Now this monitoring system has intelligent trend analysis and early warning functions. If the patient’s condition shows a fluctuating trend, it will issue an alarm in advance, allowing us to focus more on treatment.*

#### 3.1.2 Nurses hold a cautious attitude towards the human-machine collaboration model

Most emergency nurses hold a cautious acceptance attitude towards the application of the HMC Mode. They recognize its positive value in reducing workload and improving work efficiency, but also have many concerns about its reliability, safety and their own career development. Nurses generally expressed their willingness to rely on tools such as AI triage systems, central monitoring systems, and mobile nursing PDAs to assist in completing basic work, but they were cautious about the results and prompt information generated by AI. When dealing with complex cases, there is concern that the accuracy of the results generated by AI systems is hard to guarantee. Therefore, most emergency nurses will manually check and confirm the generated results to avoid clinical risks caused by AI errors.

*N9: The conditions of emergency patients change rapidly. Facing such a complex environment, I’m worried that there might be mistakes. Once a mistake occurs, it’s a big deal and we must be cautious.*

*N18: We accept human-machine collaboration with caution. It can help us reduce our burden, but it cannot replace our clinical judgment. The final decision still needs to be made by us nurses.*

### 3.2 Theme 2: role transformation of emergency nurses in HMC mode

The HMC model has driven emergency nurses to shift from experiential executors to AI-collaborative decision-makers, strengthening their role in humanistic care. However, it has also brought about practical challenges such as blurred role boundaries and expanded scope of responsibilities.

#### 3.2.1 Transform from an experience-based executor to an AI collaborative decision-maker

During the application process of the human-machine collaboration model, the role of emergency nurses has changed, gradually transitioning from traditional experience executors to AI collaborative decision-makers. The interviewed nurses indicated that before the application of the HMC Model, their clinical work was mainly based on basic operations and routine nursing execution based on clinical experience, such as manual triage, manual monitoring of vital signs, and individual information verification. The focus of their work was concentrated at the execution level. After the introduction of devices such as AI triage systems, central monitoring systems, and mobile nursing PDAs, the HMC Mode has replaced a large amount of mechanical and repetitive work for nurses, liberating them from cumbersome basic operations. Their work focus has gradually shifted to the review, analysis, judgment, and collaborative decision-making of AI output results. Nurses no longer rely solely on their own experience to complete their work. Instead, they combine the triage suggestions, risk warnings, and information prompts provided by AI, and integrate their own clinical experience with professional judgment to make comprehensive decisions on patients’ conditions and care plans.

*N8: Previously, our work mainly relied on manual triage based on experience. Now, with the AI triage system, when patients come, we input their information, and the system will provide initial suggestions. We then review the information provided by the AI. The entire process is very fast.*

*N2: Previously, ECMO patients needed us to keep a close eye on the parameters without even daring to blink. Now integrated with the intelligent system, an alarm will be triggered when the parameters are abnormal. I only need to determine whether the alarm is accurate now.*

#### 3.2.2 The humanistic care role of emergency nurses has been enhanced

The application of the human-machine collaboration model has liberated emergency nurses from a large amount of mechanical and repetitive basic work, further tilting their work focus on humanistic care and enhancing their role in humanistic care. Emergency patients are often in a state of urgent condition and anxiety, and their family members are often accompanied by panic, helplessness and other emotions. In the past, nurses spent a lot of time on basic work such as manual triage, information verification and manual monitoring of vital signs, making it difficult for them to devote sufficient energy to paying attention to the psychological state and emotional needs of patients and their families. Meanwhile, most nurses believe that the intervention of the HMC Model has saved a great deal of time and energy, enabling them to focus more on patients’ psychological comfort, emotional guidance, health advice and communication with their families. Nurses are no longer confined to performing basic nursing operations. They pay more attention to the subjective feelings of patients, actively listen to their demands, relieve their anxiety, provide psychological support and humanistic care to them, and at the same time explain the condition to the family members and soothe their emotions, building a bridge for communication between doctors and patients.

*N13: Since our department adopted the mobile nursing PDA, it has become more convenient to verify patients’ information. We now have more time to communicate with patients and patiently answer their questions.*

*N1: Now when AI helps us with triage, we can spare some time to observe patients and their families for a while longer. For those who seem very nervous or confused, we can communicate with them.*

#### 3.2.3 The boundaries of the nurse’s role are blurred

In China, since the application of the human-machine collaboration model is still in its infancy, and various operation procedures and regulations are not yet perfect, the problem of blurred role boundaries of nurses has become increasingly prominent, which has become an important predicament faced in the process of nurse role reconstruction. Before the application of the HMC Model, the work of emergency nurses was mainly concentrated in traditional nursing fields such as clinical nursing operations, disease observation, and patient care, with clear job contents and scope of responsibilities. In recent years, the application of the HMC Model has continuously expanded the job content of nurses and gradually blurred their scope of responsibilities. Nurses not only have to complete traditional nursing tasks, but also undertake the operation, maintenance, result review, and abnormal detection of AI equipment. At the same time, they also must take on the responsibility of coordination and communication between AI and patients, as well as between AI and other medical staff. Some of their work even overlaps with that of doctors and medical technicians. Furthermore, due to the lack of clear norms and responsibility divisions for human-machine collaborative work, nurses find it difficult to clearly define their core responsibilities and work boundaries in the collaborative mode. Some nurses also must undertake work beyond their professional scope, which aggravates their professional confusion and affects their role adaptation.

*N5: Sometimes our monitoring system gives false alarm warnings. I will adjust the parameters of this machine again and feel like an engineer.*

*N7: With this AI triage system, I feel more like a doctor, but when it comes to taking responsibility, I do not have the authority of a doctor.*

### 3.3 Theme 3: competency requirements of emergency nurses in human-machine collaboration mode

The HMC model place’s diverse ability demands on emergency nurses, with key areas including critical thinking on AI information, the ability to operate intelligent devices and handle emergencies, as well as awareness of data security and ethical risks.

#### 3.3.1 The demand for critical thinking ability in recognizing information output by AI

Under the HMC Mode, the demand for critical thinking ability of emergency nurses in recognizing AI output information has significantly increased, becoming one of the core capabilities for nurses to adapt to role transformation and ensure clinical safety. Due to the complexity and variability of emergency conditions and the significant individual differences among patients, although the information output by intelligent devices can provide auxiliary references, it still has limitations and may lead to problems such as inconsistency with the actual condition of the patient, deviation in early warning, and information omission. Therefore, nurses hope to enhance their critical thinking skills. When facing various types of information output by AI, they should have the ability to comprehensively review and judge the accuracy, rationality, and applicability of the information output by AI in combination with patients’ clinical symptoms, signs, and their own clinical experience, identify deviations and errors, and avoid clinical decision-making mistakes caused by blind reliance on AI information.

*N8: Once, a patient with multiple comorbidities came in. The information provided by the AI triage system was inaccurate. In the end, I made the triage based on my judgment.*

*N21: The AI of the central monitoring system indicates an abnormal indicator. I will manually monitor it again and determine whether it is truly abnormal based on the patient’s condition. We cannot be completely led by the AI.*

#### 3.3.2 The demand for intelligent device operation and emergency handling capabilities

Under the human-machine collaboration mode, intelligent devices have become important auxiliary tools for emergency nursing work. The ability of emergency nurses to operate intelligent devices and handle emergencies is the basis for ensuring the orderly progress of nursing work and avoiding equipment-related risks. The high-intensity and fast-paced nature of emergency scenarios requires nurses to be proficient in operating various intelligent devices, accurately complete information entry and parameter adjustment operations, and ensure the efficient connection between the devices and clinical nursing work. Some nurses have indicated that during daily use, smart devices may encounter various malfunctions, such as lagging of the AI triage system, failure of the central monitoring system’s early warning, signal interruption or data loss of the mobile nursing PDA, etc. If these issues cannot be dealt with promptly, they will directly affect the treatment of patients. Therefore, emergency nurses need to have strong emergency response capabilities, be able to quickly identify common equipment malfunctions, master basic troubleshooting and emergency response methods, and when equipment malfunctions occur, they should not only be able to take temporary measures promptly but also contact technicians for professional maintenance in a timely manner to avoid clinical risks caused by equipment failures.

*N17: When I first started using these devices, I wasn’t proficient enough and often made operational mistakes, which wasted a lot of time.*

*N22: Nowadays, we use mobile nursing PDAs every day at work. This requires us to be proficient in operating these devices; otherwise, we simply cannot keep up with the pace of the emergency department.*

#### 3.3.3 The need for awareness of data security and ethical risks

Under the human-machine collaboration mode, a large amount of patient personal information is generated during clinical work. These data are stored, transmitted and analyzed through human-machine collaboration devices, making nurses’ demand for data security and ethical risk awareness increasingly urgent. Patients’ personal information falls within the category of privacy. Nurses are concerned about the risk of data leakage and hope to enhance data security by strictly standardizing operation procedures, properly keeping equipment account passwords, and upgrading security systems. In addition, there are many ethical dilemmas in the process of human-machine collaboration. Some nurses are concerned about the responsibility definition of AI decision-making and patients’ informed consent for AI intervention. Therefore, nurses need to have a clear awareness of ethical risks, be able to identify and avoid ethical risks, clearly define the responsibility boundary between AI decision-making and nurses’ judgment, not blindly rely on AI, and adhere to the bottom line of nursing ethics.

*N16: All patients’ information is stored in the system. Once leaked, it not only infringes upon patients’ privacy but also incurs corresponding responsibilities. It is hoped that some measures can be taken to ensure the security of this information.*

*N6: Sometimes AI triage may have deviations. We cannot only look at the AI results but also consider the patient’s wishes and respect their choices.*

## 4 Discussion

### 4.1 Role reconstruction of emergency nurses in the HMC mode

With the application of the HMC Model in the field of emergency nursing, emergency nurses are gradually transforming from traditional experience-based nursing executors to the role of AI collaborative decision-makers. In the traditional emergency nursing model, the focus of nurses’ work is concentrated on mechanical operations such as manual triage, manual monitoring of vital signs, and repeated verification of patient information. Their professional functions are mostly limited to the implementation and execution of nursing operations, lacking the space for autonomous decision-making (16, 17). In China, with the promotion of national policies, AI technology has been gradually applied in the medical field (18). The application of intelligent devices such as AI triage systems, central monitoring systems, and mobile nursing PDAs has effectively replaced a large amount of cumbersome basic work for nurses, liberating them from the dual constraints of time and energy. Their work focus has gradually shifted to the review, analysis, and comprehensive decision-making of AI output information. Watson et al. (19) explored the application of AI in clinical nursing, further confirming that AI can reduce the workload of nurses and is undergoing a higher-level transformation from basic tasks to AI collaboration. In addition, during the process of character transformation, there is also the realistic predicament of blurred character boundaries. In China, due to the lack of relevant policies and regulations, a clear system of responsibility division and operational norms have not yet been formed. Unlike countries in Europe and America where medical informatization is mature, the human-machine collaboration model in China is still in the exploration stage in the field of emergency care (8). Against this backdrop, nurses not only need to complete traditional nursing operations, review the output results of AI, and correct AI biases, but also need to coordinate well between AI decisions and the subjective wishes of patients. They should not only explain the principles and limitations of AI intervention to patients and strive for their cooperation, but also make reasonable judgments based on AI results and the actual needs of patients, balancing the human-machine collaboration mode The need for humanistic care. Tuncer et al. (20) conducted qualitative research interviews with 28 nurses. This study revealed that in the AI era, nurses can provide more humanistic care for patients and have irreplaceable value. Under the current human-machine collaboration model, nurses frequently switch between multiple roles such as traditional nursing executors, AI reviewers, and clinical decision-makers, which makes nurses lack a clear understanding and positioning of their own roles and may subsequently lead to cognitive confusion and job burnout. Therefore, improving relevant policies and regulations is of vital importance for the role reconstruction of emergency department nurses in the HMC Mode.

### 4.2 The current situation and demands of emergency nurses in the HMC mode

Emergency nurses’ understanding of the human-machine collaboration model shows recognition of its value, but at the same time they maintain a cautious attitude in application. This current situation demonstrates the needs of nurses in terms of ability and support. In this study, the working years of nurses ranged significantly (from 1 to 21 years), but the positive role of the HMC model in reducing workload, improving the efficiency of emergency triage, and optimizing disease early warning capabilities was generally recognized. This might be related to the fact that AI devices have been popularized in the emergency department for a relatively short period of time in recent years. Both senior and junior nurses are using human-machine collaborative systems at the same time. Meanwhile, junior nurses often possess better digital literacy, while senior nurses have more extensive clinical judgment experience. They can better integrate intelligent devices into the entire process of emergency care, including pre-examination and triage, disease monitoring, and information verification, thus becoming an important auxiliary tool for nurses to carry out their work. Blaschke et al. (21) conducted a multi-center randomized controlled trial in eight emergency departments and found that under the HMC Model, the AI-assisted triage system could reduce the average waiting time for patients by 20 min, alleviating the triage pressure on nurses. But at the same time, nurses remain highly cautious about the reliability and safety of AI technology. The human-machine collaboration model has obvious limitations in clinical applications, such as the mismatch between AI triage and the actual condition of patients, deviations in early warnings from the central monitoring system, and information omissions from mobile nursing PDAs. These issues require nurses to combine patients’ subjective wishes and clinical experience in clinical practice to correct AI deviations and ensure the safety of patients’ diagnosis and treatment. This is consistent with the research results of Morey et al. (22). Therefore, emergency nurses have shown demands for critical thinking, emergency handling capabilities, ethical risk awareness, etc. in the HMC Mode. In this study, the nurses’ demand for critical thinking ability is mainly manifested in that when facing the information output by AI, problems such as deviations may occur. Nurses need to have independent clinical judgment ability and conduct a comprehensive review and correction of the AI output results in combination with the patients’ symptoms, medical history and subjective will. Secondly, nurses have a strong demand for the operation and emergency handling capabilities of intelligent devices. Currently, the research and development of some emergency intelligent devices focuses on technological advancement, while neglecting the clinical operation habits of nurses and the fast-paced work characteristics of emergency departments. The complexity of device operation is relatively high, and the emergency response to faults is not timely, which forces nurses to invest additional energy in mastering the operation and emergency handling skills of the devices. Li et al. (23) found that nurses are confronted with the challenge of complex human-computer interaction functions in clinical practice. Nurses not only need to deal with equipment alarms but also face inconsistent practice guidelines, thus having an urgent need for the ease of operation of human-computer interaction. In addition, the demands of nurses in the HMC Mode are also reflected in their awareness of data security and ethical risks. In China, with the popularization of smart devices in emergency departments, the number of links in the storage, transmission and application of patients’ private data has increased. However, the relevant norms are not yet perfect, which makes nurses must prevent data leakage and abuse as well as deal with the ethical dilemmas brought about by AI decision-making. It is hoped that the burden beyond the scope of duties can be reduced through the improvement of the system.

### 4.3 Optimization and guarantee of human-machine collaboration mode in emergency nursing field

The optimization and guarantee of the HMC Model in the field of emergency nursing should adhere to the principle of adapting technology to clinical practice and ensuring safety through systems, to improve the quality of nursing while considering the rights and interests of patients and the career development of nurses. At the technical level, the focus should be on the adaptability of AI technology in emergency scenarios. In response to issues such as AI triage bias and false alarms in early warning and considering the complex conditions and significant individual differences of emergency patients, the AI algorithm should be optimized, and the linkage mechanism among the AI triage system, central monitoring system, and mobile nursing PDA should be improved to enhance the accuracy and pertinence of the AI output information. Reduce the review burden on nurses while retaining their authority to correct AI results and fully leverage the clinical leading role of nurses. Lansiaux et al. (24) compared three AI triage models and found that there were significant deviations among different models. Regularization, external validation, and security assessment need to be conducted before deployment to enhance accuracy and security. At the level of capacity guarantee, it is necessary to build a nurse capacity training system in a stratified and classified manner. Based on the capacity shortcomings of junior, intermediate and senior nurses, targeted training on AI information recognition, intelligent equipment operation, emergency handling, data security and ethical norms should be carried out to strengthen nurses’ critical thinking and clinical judgment abilities, helping them quickly adapt to the needs of role transformation. Nursing managers need to clarify the job responsibilities and work boundaries of emergency nurses under the human-machine collaboration mode, formulate standardized work processes, standardize the responsibilities of nurses in aspects such as AI result review, abnormal early warning, and equipment usage, and reduce role confusion and additional burdens. Establish a multi-disciplinary collaboration and division of labor mechanism, and clearly define the responsibilities of nursing, medical, information technology and equipment maintenance personnel. At the same time, improve relevant management systems and ethical norms, clarify the responsibility division when AI-assisted decision-making fails, and reduce the professional pressure on nurses. In addition, it is necessary to establish and improve the professional security mechanism for nurses, pay attention to the professional confusion of nurses during the process of role reconstruction, optimize the work process, reduce the additional workload of nurses, enhance their professional identity and job satisfaction, and promote the standardization and sustainable development of the HMC model in the field of emergency nursing.

## 5 Limitations

This study explored the role reconstruction and capability requirements of emergency nurses under the HMC Model through qualitative research methods, providing a reference for clinical practice in this field. However, there are still certain limitations. First, the selection of research subjects is subject to geographical and institutional type limitations. This study only selected emergency nurses from two tertiary hospitals in Shanghai as the research subjects. All the research subjects were from high-level medical institutions in economically developed areas, where the configuration of human-machine collaborative equipment was more complete, and the nurses had richer experience in using intelligent devices. However, there may be differences in the degree of human-machine collaborative application, nurses’ experience and demands in primary hospitals and medical institutions in economically underdeveloped areas in China. Second, the research was designed as a qualitative study. Data collection was carried out through semi-structured interviews. During the interviews, no relevant data such as the respondents’ body language and expressions were collected, which might affect the data processing process. Meanwhile, only the subjective experiences and demands of emergency nurses at the current development stage of the human-machine collaboration model have been captured, and it is impossible to dynamically track the changing trends of nurses’ role reconstruction and ability demands during the iteration of human-machine collaboration technology and the improvement of policy systems. Finally, the perspective of this study is the nurse population, and the sample size is relatively small, not including doctors, hospital administrators, and AI technology developers, which may lead to incomplete research results. Therefore, in the future, the sample size in different regions should be expanded, and multi-angle explorations should be conducted on the role reconstruction and capability requirements of emergency nurses in the human-machine collaboration mode from the perspectives of doctors, hospital administrators, and technical developers.

## 6 Conclusion

This study explores the changes in the role reconstruction and capability requirements of emergency nurses in the HMC Model in the context of China. It has clarified the nurses’ recognition and cautious acceptance of the value of the HMC Model, promoting the transformation of nurses from traditional experience-based nursing executors to AI collaborative decision-makers. By analyzing the views on the role reconstruction process of emergency nurses in the human-computer collaboration mode, the challenges faced by emergency nurses in the human-computer collaboration mode are revealed. In the future, targeted ability training programs, role norms and management systems can be developed based on research results, and clinical applications and effect evaluations can be carried out.

## Data Availability

The original contributions presented in the study are included in the article/Supplementary material, further inquiries can be directed to the corresponding authors.
