# Supplementary material for: Role reconstruction and competency requirements of emergency nurses in the human-machine collaborative mode: a qualitative study
Source: Front Public Health. 2026 Apr 23;14:1831771. doi: 10.3389/fpubh.2026.1831771 (PMC13149459; doi:10.3389/fpubh.2026.1831771)
Supplement: Supplementary file 1 [file Table_1.DOCX]

**Supplementary materials**

**S1: General Information**

| Number | Age | Gender | Education | Professional Title | Emergency working years | Job position | Usage time of AI devices | Frequency | Commonly Used equipment |
| --- | --- | --- | --- | --- | --- | --- | --- | --- | --- |
| N1 | 28 | Female | Undergraduate | Junior | 5 | Triage | 2 | Every day | AI triage system |
| N2 | 34 | Female | Master's degree | Intermediate | 11 | Resuscitation room | 3 | Every day | Central Guardianship system |
| N3 | 26 | Female | Undergraduate | Junior | 3 | Infusion room | 1 | Every day | Mobile nursing PDA |
| N4 | 41 | Female | Junior college | Intermediate | 18 | Resuscitation room | 3 | Every day | Central Guardianship system |
| N5 | 30 | Male | Undergraduate | Junior | 6 | Resuscitation room | 3 | Every day | Central Guardianship system |
| N6 | 45 | Female | Junior college | Intermediate | 21 | Triage | 2 | Every day | AI triage system |
| N7 | 23 | Female | Undergraduate | Junior | 1 | Triage | 0.5 | Every day | AI triage system |
| N8 | 32 | Female | Undergraduate | Intermediate | 8 | Triage | 1 | Every day | AI triage system |
| N9 | 29 | Female | Undergraduate | Junior | 5 | Resuscitation room | 1 | Every day | Central Guardianship system |
| N10 | 35 | Female | Junior college | Junior | 11 | Infusion room | 1 | Every day | Mobile nursing PDA |
| N11 | 25 | Female | Undergraduate | Junior | 1 | Infusion room | 0.5 | Every day | Mobile nursing PDA |
| N12 | 33 | Male | Undergraduate | Junior | 9 | ICU | 3 | Every day | Central Guardianship system |
| N13 | 41 | Female | Undergraduate | Intermediate | 17 | Emergency ward | 1 | Every day | Mobile nursing PDA |
| N14 | 29 | Female | Master's degree | Junior | 3 | Resuscitation room | 2 | Every day | Central Guardianship system |
| N15 | 24 | Male | Junior college | Junior | 1 | Resuscitation room | 1 | Every day | Central Guardianship system |
| N16 | 31 | Female | Junior college | Junior | 8 | Emergency ward | 1 | Every day | Mobile nursing PDA |
| N17 | 39 | Female | Undergraduate | Intermediate | 15 | ICU | 3 | Every day | Central Guardianship system |
| N18 | 31 | Female | Undergraduate | Junior | 7 | Triage | 1 | Every day | AI triage system |
| N19 | 26 | Female | Junior college | Junior | 4 | Infusion room | 1 | Every day | Mobile nursing PDA |
| N20 | 27 | Male | Undergraduate | Junior | 4 | ICU | 3 | Every day | Central Guardianship system |
| N21 | 36 | Female | Master's degree | Intermediate | 13 | ICU | 3 | Every day | Central Guardianship system |
| N22 | 30 | Female | Undergraduate | Junior | 5 | Emergency ward | 1 | Every day | Mobile nursing PDA |
